# Associations of executive function and age of first use of methamphetamine with methamphetamine relapse

**DOI:** 10.3389/fpsyt.2022.971825

**Published:** 2022-10-13

**Authors:** Lin-Lin Mu, Yan Wang, Li-Jin Wang, Ling-Ling Xia, Wei Zhao, Pei-Pei Song, Jun-Da Li, Wen-Juan Wang, Lin Zhu, Hao-Nan Li, Yu-Jing Wang, Hua-Jun Tang, Lei Zhang, Xun Song, Wen-Yi Shao, Xiao-Chu Zhang, Hua-Shan Xu, Dong-Liang Jiao

**Affiliations:** ^1^School of Mental Health, Bengbu Medical College, Bengbu, China; ^2^Compulsory Isolated Drug Rehabilitation Center, Bengbu, China; ^3^Chinese Academy of Sciences (CAS) Key Laboratory of Brain Function and Disease and School of Life Sciences, University of Science and Technology of China, Hefei, China

**Keywords:** methamphetamine use disorder, executive function, mental disorders, the age of first use, relapse

## Abstract

**Background and aims:**

Methamphetamine (MA) is a psychostimulant associated with a high relapse rate among patients with MA use disorder (MUD). Long-term use of MA is associated with mental disorders, executive dysfunction, aggressive behaviors, and impulsivity among patients with MUD. However, identifying which factors may be more closely associated with relapse has not been investigated. Thus, we aimed to investigate the psychological factors and the history of MA use that may influence MA relapse.

**Methods:**

This cross-sectional study included 168 male MUD patients (MUD group) and 65 healthy male residents (control group). Each patient was evaluated with self-report measures of executive dysfunction, psychopathological symptoms, impulsiveness, aggressiveness, and history of MA use. Data were analyzed with *t*-tests, analyses of variance, and correlation and regression analyses.

**Results:**

The MUD group reported greater executive dysfunction, psychopathological symptoms, impulsivity, and aggression than the control group. Lower age of first MA use was associated both with having relapsed one or more times and with having relapsed two or more times; greater executive dysfunction was associated only with having relapsed two or more times.

**Conclusion:**

Patients with MUD reported worse executive function and mental health. Current results also suggest that lower age of first MA use may influence relapse rate in general, while executive dysfunction may influence repeated relapse in particular. The present results add to the literature concerning factors that may increase the risk of relapse in individuals with MUD.

## Introduction

Addiction to amphetamine-type stimulants is a global public health issue. According to the World Drug Report 2022 (1), methamphetamine (MA) is commonly used substance among amphetamine-type stimulants and widely used drug in China. By the end of 2021, China had about 0.79 million MA users, accounting for 53.4% of the total number of drug use disorders (2). MA use disorders (MUDs) is equivalent to the DSM-5 term of amphetamine-type substance use disorder that is a subtype of stimulant use disorders (3). MUD is any form of chronic and problematic MA use including abuse, misuse, dependence and use disorder regarding MA (4).

Studies have suggested that MA is highly addictive with a high relapse rate (5). However, there is a lack of effective methods to detect and reduce the likelihood of relapse.

Studies have observed an increased likelihood of mental disorders and cognitive impairment among individuals who use MA (6), with estimates that between 40 and 60% of users are thus affected (7, 8). The mental disorder symptoms include depression, anxiety, irritability, violent behavior, hallucinations, and delusions (9–11), while cognitive impairment includes deficits in learning, memory, attention, decision-making, social cognition, executive function, and working memory (12, 13). Such symptoms often produce progressive social and occupational deterioration as well as poor treatment outcomes, and some of these psychological indicators are closely related to relapse. For example, it has been found that treating depression and anxiety plays a vital role in preventing relapse in MUD patients (14). Impulsive behavior has been associated with the severity of MA addiction, and it can be used to predict MUD patients' quality of life following treatment (15). MA-induced aggressive behavior has also been associated with MUD relapse (10).

According to the research, executive function plays a crucial role in the prognosis of treatment efficacy and in preventing relapse in addiction, suggesting that improvement of MUD patients' executive function may enhance the effectiveness of their treatment (16). Executive function is an umbrella term that includes cognitive processes such as decision-making, impulse control, inhibitory control, behavioral flexibility, and working memory. Good executive function can identify and effectively control impulsive and compulsive drug-seeking behavior, thereby reducing the likelihood of relapse (17). Therefore, we suspected that executive dysfunction and related factors, including psychopathological symptoms, impulsivity, and aggression, may play a role in MA relapse.

Based on previous findings, the present study compared adult patients with MUD to healthy adults with no history of MA use in relation to executive dysfunction, psychopathological symptoms, impulsiveness, and aggressiveness. In an attempt to expand on the literature, the present study also aimed to investigate the psychological factors and the history of MA use that may influence MA relapse. Specifically, a key aim of the current study was to try to identify which factors (e.g., psychopathological symptoms, impulsive/aggressive traits, and MA usage characteristics) may be more closely associated with MA relapse.

## Materials and methods

### Subjects

A cross-sectional design was used in the current study. Male MUD patients (*n* = 168) were recruited from Bengbu Compulsory Isolated Drug Rehabilitation Center from July 2019 to March 2021. All participants met DSM-5 criteria for stimulant use disorder (methamphetamine-type), which will be referred to as MUD in this report. The diagnosis was confirmed by an associate professor psychiatrist. Inclusion criteria: (1) between 18 and 45 years old; (2) normal vision and hearing; (3) more than 6 years of education, i.e., primary school level or above; (4) participation in MA withdrawal treatment for < 3 months; (5) no other substance use disorder (e.g., opioids, cocaine, or alcohol, except for cigarettes) in the past 5 years. Exclusion criteria: (1) mental disorders or neurological diseases (e.g., schizophrenia, mood disorder, stroke, epilepsy, or Parkinson's disease); (2) other chronic diseases (e.g., diabetes, hypertension, hyperlipidemia, and gastrointestinal diseases); (3) using any medication which may affect cognitive and executive function.

The staff members of the Bengbu Mental Health Center and the people in the local community were chosen as the control group (65 healthy adults), and none of them had a history of illicit drug use. The control parameters, such as gender, age, and education, matched the MA groups. All participants had to sign an informed consent form as a protocol. The study was approved by the Institutional Review Board (permission number: 2017-53) of Bengbu Medical University. All experiments were carried out following the approved guidelines and regulations.

### Tools

#### Demographic questionnaire

This was used to collect the demographic information of the MA and Control groups, including age, years of education, and marital status.

#### Drug use questionnaire

Information on drug use by MUD patients was collected, including the age of first MA use (years), total duration of MA use (months), MA use before abstinence (g/occasion), and the number of relapses (times). The number of relapses was represented by the number of times MUD patients entered the Compulsory Isolated Drug Rehabilitation Center.

#### Behavior rating inventory of executive function-adult version (BRIEF-A)

The BRIEF-A is a clinically validated questionnaire of executive function consisting of nine subscales (Inhibit, Self-Monitor, Plan/Organize, Shift, Initiate, Task Monitor, Emotional Control, Working Memory, and Organization of Materials) tapping into various aspects of executive functioning in daily life (18). The BRIEF-A total score (an overall score that summarizes the nine subscales) is known as the Global Executive Composite (GEC). The BRIEF-A has 75 items on a three-point scale. Higher scores denote more impaired executive function. In this study, internal consistency of Cronbach's α of the questionnaire was 0.956, indicating that the scale had good reliability.

#### Self-report symptom inventory, symptom checklist 90 (SCL-90)

The SCL-90 (19) is a 90-item, five-point scale inventory used to evaluate psychopathological symptoms. The SCL-90 measures nine symptom domains of psychological distress: somatization, obsessive compulsion, interpersonal sensitivity, depression, anxiety, hostility, phobic anxiety, paranoid ideation, psychoticism, and “additional items.” This study includes 10 subscales and the Global Severity Index (GSI). Cronbach's α of the questionnaire measured internal consistency was 0.907, and internal consistency by Cronbach's α of subscales was 0.716–0.857, indicating that the scale had good reliability.

#### Barratt impulsiveness scale 11 (BIS-11)

The BIS-11 (20) is used to evaluate the impulsive characteristics of individuals. The BIS-11 has 30 items spanning three dimensions: attentive impulse, motor impulse, and non-planning impulse. Each item is scored with a five-point scale. Higher scores reflect higher impulsivity and hyperactivity, inattention, and lack of planning. In this study, internal consistency was measured by Cronbach's α of the questionnaire was 0.887, indicating that the scale had good reliability.

#### Chinese version of buss-perry aggression questionnaire (AQ-CV)

The AQ-CV (21) is used to evaluate the aggressiveness of the subjects. The AQ-CV has 30 items assessing five dimensions of aggression: Physical Aggression, Verbal Aggression, Anger, Hostility, and Self-Aggression. Each item is scored with a five-point scale. A higher total score reflects higher aggression and aggressive traits. In this study, internal consistency measured by Cronbach's α of the questionnaire was 0.907, indicating that the scale had good reliability.

### Statistical analysis

The SPSS 25.0 software (IBM Corporation, Armonk, NY, USA) was used for statistical analysis in this study. The measured data were expressed as (mean ± standard deviation, M ± SD), and the independent sample *t*-test was used to compare two groups of measured data. 2-Sample *t*-test, α = 0.05, power values > 0.8, the sample size was calculated and compared with the actual sample size, if the calculated sample size was lower than the actual sample size, it passed the power analysis. Power calculations were conducted using minitab using a type 1 error rate (α) = 0.05, power (1 –β) = 0.80, effect size: Cohen's d (Cohen's *d* > 0.5, medium), which recommended a total sample size of N = 300 (MA group:216; Control group: 84). One-way Analysis of Variance (ANOVA) or Fisher's exact tests with Bonferroni *post-hoc* measured data for multiple groups, making multiple comparisons. Also, Spearman correlation analyses were used to identify the relationships between information on MA use and psychological characteristics. To correct for multiple comparisons, a *p*-value of 0.05/21 = 0.0024 was deemed significant. Ordinal regressions were used to assess the demographic information and psychological scale scores of MUD patients with varying the number of relapses. Binary logistic regression analysis was used to construct the prediction model equation of MA relapse. Discrimination and calibration of prediction models were tested using the receiver operating characteristic (ROC) curve test and the Hosmer-Lemeshow test. *P*-values < 0.05 (two-sided tests) were considered statistically significant.

## Results

### Demographics, MA use history in MA group and control group

There was no significant difference in age, education years, and marital status between the MA and Control groups (*P* > 0.05). In the MA group, first MA use was at 25.77 ± 7.44 years, total duration of MA use was 118.58 ± 72.22 months, MA use before abstinence was 0.49 ± 0.38 g/occasion, and the number of relapses was 2.13 ± 0.99 times (see Table 1).

**Table 1:** Demographics and history of MA use in the MA group and control group.

	**MA group (*n* = 168)**	**Control group (*n* = 65)**	***t*/*x*^2^**	***P***
Age (years)	34.27 ± 6.60	34.15 ± 6.32	0.126	0.900
Education year (years)	6.94 ± 2.88	7.55 ± 2.70	−1.741	0.143
**Marital status**				
Married (%)	92 (54.76%)	36 (55.39%)		
Unmarried (%)	38 (22.62%)	14 (21.54%)	0.034	0.984
Divorced (%)	38 (22.62%)	15 (23.07%)		
Widowed (%)	0	0		
The age of first MA use (years)	25.77 ± 7.44			
Total duration of MA use (months)	118.58 ± 72.22			
MA use before abstinence (g/occasion)	0.49 ± 0.38			
Number of relapses (times)	1.54 ± 0.27			

Data accord with normal distribution were given as mean ± standard deviation (M ± SD).

MUD, methamphetamine use disorder.

### Comparison of psychological characteristics between the MA and control groups

Independent-sample *t*-tests were used to compare the BRIEF-A, BIS-11, and AQ-CV total scores and the SCL-90 subscale scores between the MA and control groups. MUD patients reported greater executive dysfunction, impulsiveness, aggressiveness, and psychopathological symptoms relative to the control group (see Table 2). All the variables with significant differences passed the power analysis.

**Table 2:** Comparison of psychological characteristics between the MA group and control group.

**Variable**	**MA group (*n* = 168)**	**Control group (*n* = 65)**	***t***	***P***
GEC (BRIEF-A total score)	106.92 ± 23.54	97.91 ± 25.04	2.575	0.011*
**SCL-90**				
Somatization	21.79 ± 8.05	15.26 ± 5.72	6.846	0.000***
Obsessive compulsion	20.75 ± 7.03	20.35 ± 5.79	0.407	0.685
Interpersonal sensitivity	15.79 ± 5.82	15.86 ± 6.34	−0.079	0.937
Depression	23.66 ± 8.72	20.57 ± 8.09	2.459	0.015*
Anxiety	17.32 ± 6.68	15.11 ± 6.11	2.305	0.022*
Hostility	10.70 ± 4.64	8.72 ± 3.26	3.127	0.002**
Phobic anxiety	9.72 ± 3.51	10.12 ± 3.92	−0.759	0.448
Paranoid ideation	9.76 ± 3.74	9.00 ± 3.24	1.434	0.153
Psychoticism	16.40 ± 6.08	15.23 ± 5.86	1.324	0.187
Additional items	12.92 ± 4.35	10.06 ± 3.90	4.602	0.000***
GSI (SCL-90 total score)	158.66 ± 50.94	158.66 ± 46.71	2.525	0.012*
BIS-11 total score	44.57 ± 15.08	37.45 ± 12.27	3.395	0.001**
AQ-CV total score	37.52 ± 17.63	27.33 ± 14.30	4.558	0.000***

Data accord with normal distribution were given as mean ± standard deviation (M ± SD).

BRIEF-A, Behavior Rating Inventory for Executive Function of adult version; SCL-90, Self-report symptom inventory, Symptom checklist 90; GEC, Global Executive Composite; GSI, Global Severity Index; BIS-11, Barratt Impulsiveness Scale-11; AQ-CV, Chinese version of Buss-Perry aggression questionnaire.

*P < 0.05, **P < 0.01, ***P < 0.001.

### Relationship between MA use history and psychological characteristics of MUD patients

Spearman correlation analyses were used to identify the relationships between the information on MA use history and psychological characteristics in the MA group.

After Bonferroni's corrections, there's a correlation between number of relapses and the age of first MA use, between age and the age of first MA use, between age and total duration of MA use. None of the other scores and sub-scales showed statistically significant correlations (*P* > 0.0023; see Table 3).

**Table 3:** Relationship between MA use history and psychological characteristics of MUD patients.

**Variable**	**Number of relapses (times)**	**The age of first MA use (years)**	**Total duration of MA use (months)**	**MA use before abstinence (g/occasion)**
	***r***	***r***	***r***	***r***
Age (years)	0.118	0.694***	0.303***	0.012
Marital status	0.073	−0.193*	0.114	0.104
Education year (years)	0.080	−0.215**	0.009	0.023
Number of relapses (times)	1	−0.274***	0.187*	0.118
The age of first MA use (years)	−0.274***	1	−0.224**	−0.219**
Total duration of MA use (months)	0.187*	−0.224**	1	0.212**
MA use before abstinence (g/occasion)	0.118	−0.219**	0.212**	1
GEC (BRIEF-A total score)	0.225**	−0.075	0.106	0.098
**SCL-90**				
Somatization	0.015	0.003	0.140	−0.013
Obsessive compulsion	0.007	0.041	0.083	0.007
Interpersonal sensitivity	0.059	0.029	0.070	−0.009
Depression	0.007	0.093	0.116	−0.062
Anxiety	0.004	0.127	0.038	−0.026
Hostility	0.071	0.014	0.017	0.019
Phobic anxiety	0.059	−0.016	0.020	0.104
Paranoid ideation	0.190*	0.026	0.035	−0.014
Psychoticism	0.037	0.121	0.091	−0.067
Additional items	0.075	0.039	0.047	−0.051
GSI (SCL-90 total score)	0.034	0.058	0.098	−0.010
BIS-11 total score	0.155*	−0.153*	0.083	0.111
AQ-CV total score	0.148	−0.076	0.118	0.077

MUD, methamphetamine use disorder; BRIEF-A, Behavior Rating Inventory for Executive Function of adult version; BIS-11, Barratt Impulsiveness Scale-11; GEC, Global Executive Composite; GSI, Global Severity Index; SCL-90, Self-report symptom inventory; AQ-CV, Chinese version of Buss-Perry aggression questionnaire.

*P < 0.05, **P < 0.01, Bonferroni's corrections, ***P < 0.05/21 = 0.0023.

### Comparison of demographic information and psychological characteristics in MUD patients with different number of relapses

The demographic information and psychological assessment scores of MUD patients with varying the number of relapses were compared. The age of first MA use, total duration of MA, phobic anxiety, AQ-CV total score, BIS-11 total score, and GEC were significantly different with different number of relapses (*P* < 0.05), based on one-way ANOVA or Fisher's exact tests with Bonferroni *post-hoc* analysis, as shown in Table 4.

**Table 4:** Comparison of demographic information and psychological characteristics in MUD patients with different number of relapses.

	**Number of relapses**			
	**Zero^1^ (*n* = 46)**	**Once^2^ (*n* = 73)**	**Twice and more relapse^3^ (*n* = 49)**	***F*/*x*^2^**	***P***	***Post-hoc***
Age (years)	33.23 ± 7.80	34.23 ± 5.74	35.12 ± 6.48	0.955	0.387	
Education year (years)	6.89 ± 3.72	6.59 ± 2.33	7.51 ± 2.63	1.392	0.251	
Marital status	1.52 ± 0.69	1.73 ± 0.85	1.87 ± 0.94	1.569	0.199	
The age of first MA use (years)	28.5 ± 8.73	25.52 ± 5.73	23.15 ± 7.07	6.570	0.002**	1>2, 1>3
First MA use occurred at 19 years old or younger (%)	6 (13.04)	12 (16.43)	22 (44.89)	17.138	0.000**	3>2, 3>1
Total duration of MA use (months)	102.23 ± 74.68	114.48 ± 65.80	140.94 ± 75.33	3.661	0.028*	1 < 3, 2 < 3
MA use before abstinence (g/occasion)	0.48 ± 0.32	0.43 ± 0.43	0.58 ± 0.32	2.199	0.114	
GEC (BRIEF-A total score)	104.9 ± 21.88	103.85 ± 22.55	114.85 ± 25.65	3.857	0.023*	1 < 3, 2 < 3
**SCL-90**						
Somatization	22.16 ± 8.70	21.46 ± 7.56	21.93 ± 8.26	0.116	0.891	
Obsessive compulsion	21.50 ± 6.30	20.25 ± 7.26	20.80 ± 7.41	0.379	0.685	
Interpersonal sensitivity	16.16 ± 5.6	15.28 ± 5.84	16.21 ± 5.10	0.548	0.579	
Depression	24.80 ± 8.93	22.94 ± 8.6	23.65 ± 8.19	0.728	0.485	
Anxiety	18.68 ± 7.69	16.29 ± 6.18	17.57 ± 6.25	2.054	0.132	
Hostility	11.00 ± 4.80	9.94 ± 4.13	11.54 ± 5.11	1.697	0.187	
Phobic anxiety	9.86 ± 3.68	9.43 ± 3.03	10.00 ± 4.02	0.464	0.629	
Paranoid ideation	10.72 ± 4.43	8.99 ± 3.17	10.00 ± 3.66	3.079	0.049*	2 < 1
Psychoticism	17.07 ± 6.01	15.68 ± 6.03	16.84 ± 6.21	0.907	0.406	
Additional items	13.23 ± 4.79	12.42 ± 3.93	13.39 ± 4.51	1.021	0.363	
GSI (SCL-90 total score)	162.98 ± 51.59	154.18 ± 50.23	160.88 ± 42.75	0.541	0.583	
BIS-11 total score	43.58 ± 13.29	41.80 ± 14.79	48.62 ± 15.33	3.654	0.028*	2 < 3
AQ-CV total score	38.04 ± 15.86	33.96 ± 16.68	42.91 ± 19.62	3.421	0.035*	2 < 3

One-way ANOVA or Fisher's exact tests with Bonferroni *post-hoc* analysis, test level after correction for multiple comparisons is *P* = 0.05/3 = 0.017. Only the age of first MA use were significantly different with different number of relapses (*P* < 0.017). *P < 0.05; **P < 0.017.

MUD, methamphetamine use disorder; BRIEF-A, Behavior Rating Inventory for Executive Function of adult version; GEC, Global Executive Composite; SCL-90, Self-report symptom inventory, Symptom checklist 90; GSI, Global Severity Index; BIS-11, Barratt Impulsiveness Scale-11; AQ-CV, Chinese version of Buss-Perry aggression questionnaire.

Test level after correction for multiple comparisons is *P* = 0.05/3 = 0.017. Only the age of first MA use and first MA use occurred at 19 years old or younger were significantly different with different number of relapses (*P* < 0.017).

### The influencing factors of the number of relapses were identified using ordinal regression analysis

In order to conduct an ordinal regression analysis, the variables (*P* < 0.05) in Table 4 were used as independent variables and the number of relapses as dependent variables. The results showed that the age of first MA use, the total scores of BRIEF-A (GEC), and BIS-11 entered the regression equation (see Table 5). The parallel line test *P* = 0.124 > 0.05 indicates no multicollinearity between variables of the regression equation.

**Table 5:** Using ordinal regression analysis to screen the influencing factors of the number of relapses.

**Variable**	**Estimate**	**S.E**	**Wald**	***P***	***VIF***
The age of first MA use (years)	−0.068	0.023	8.829	0.003**	1.083
Total duration of MA use (months)	0.003	0.002	2.140	0.143	1.063
Paranoid ideation	−0.009	0.013	0.412	0.521	1.616
AQ-CV Total score	0.013	0.012	1.223	0.269	1.815
BIS-11 total score	0.025	0.010	6.289	0.012*	2.203
GEC (BRIEF-A total score)	−0.163	0.059	7.701	0.006**	1.856

AQ-CV, Chinese version of Buss-Perry aggression questionnaire; BRIEF-A, Behavior Rating Inventory for Executive Function of adult version; GEC, Global Executive Composite; BIS-11, Barratt Impulsiveness Scale-11.

The parallel line test *P* = 0.124 > 0.05 indicates that there is no multicollinearity.

*P < 0.05, **P < 0.01.

### Construction of prediction model for MA relapse by binary logistics regression analysis

Among 168 MUD patients, 46 patients had never relapsed (zero relapse), 73 patients had relapsed one time (one relapse), and 49 patients had relapsed two or more (≥ two relapses). Considering (1) zero relapse (46 patients) and ≥ one relapse (122 patients) and (2) ≤ one relapse (119 patients) and ≥ two relapses (49 patients) as the dependent variables and the age of first MA use, BIS-11 total score, and BRIEF-A total score (GEC) as the independent variables, binary logistic regression analyses were conducted to construct the two relapse prediction model equations (see Table 6). The two prediction model equations showed that the age of first MA use was a significant predictor of ≥ one relapse and ≥ two relapses; GEC (executive dysfunction) was a significant predictor of ≥ two relapses; and BIS-11 total score was not a significant predictor in either relapse prediction model.

**Table 6:** Construction of prediction model for MA relapse using binary logistic regression analysis.

**Construction of relapse prediction model**	**Dependent variable**	**0 Relapse (46 patients) and** ≥**once relapse (122 patients)**				≤ **Once relapse (119 patients) and** ≥**twice relapse (49 patients)**			
	**Independent variable**	**The age of first MA use (years)**	**GEC**	**BIS-11 total score**	**Constant**	**The age of first MA use (years)**	**GEC**	**BIS-11 total score**	**Constant**
	B	−0.070	0.009	−0.010	2.264	−0.069	0.027	−0.092	−1.267
	S.E	0.024	0.009	0.012	1.084	0.030	0.010	0.068	1.152
	Wald	8.435	0.916	0.755	4.361	5.184	6.921	1.837	1.209
	*P*	0.004**	0.339	0.385	0.037	0.023*	0.009**	0.175	0.272
	Exp(B)	0.932	1.009	0.990	9.624	0.934	1.028	0.912	0.282
	Relapse model	Equation one = 2.264–0.070* The age of first MA use				Equation two = −1.267–0.069* The age of first MA use + 0.027*GEC			
Discrimination	ROC curve	AUC = 0.650 95% Cl (0.555–0.745) *P* = 0.003				AUC = 0.669 95% Cl (0.577–0.761) *P* = 0.001			
Calibration	Hosmer-Lemeshow	*R*^2^ = 11.273, *P* = 0.187				*R*^2^ = 12.091, *P* = 0.147			

The prediction models of MA relapse were constructed by binary logistics regression analysis. The variable that predicts ≥ once relapse is age of first MA use. The variables that predicts ≥ two times relapse are age of first MA use and GEC. ROC curve tests and Hosmer-Lemeshow test demonstrated that the discrimination and calibration of two relapse model equations were all very high.

GEC, Global Executive Composite; BIS-11, Barratt Impulsiveness Scale-11; AUC, area under the curve; CI, confidence interval.

*P < 0.05, **P < 0.01.

## Discussion

Current results indicated that MUD patients had greater executive dysfunction, psychopathological symptoms, impulsiveness, and aggressiveness than healthy controls. Previous studies also found that MUD patients exhibit executive dysfunction, anxiety, depression, impulsive behavior, and aggressiveness (9, 10, 12, 22). Furthermore, in the current study, lower age of first MA use was associated both with having relapsed one or more times and with having relapsed two or more times, whereas greater executive dysfunction was associated only with having relapsed two or more times. Hence, current findings further suggest that lower age of first MA use may influence relapse rate in general, while greater executive dysfunction may influence higher rates of relapse in particular.

1. Executive dysfunction is associated with relapse.

Executive function is often viewed as a complex cognitive function that includes a series of functions such as inhibition, working memory, planning, impulse control, mental flexibility, and initiating and monitoring actions (23). Specifically, the most important executive function factor related to relapse is inhibitory control (24). Drug addiction can be viewed as a transition from voluntary, recreational drug use in the early stages to habitual and compulsive drug-seeking in the later stages (25, 26). Habitual drug use was the basis of compulsive drug-seeking. In habitual phase, when drugs are not available, addicts experience strong cravings, leading to the transformation of the habit into compulsive drug-seeking behaviors or relapse (27). Compulsive drug-seeking behaviors and relapse can be defined as the maladaptive persistence of response despite adverse consequences (28) and represents a loss of top-down inhibitory control (29, 30). Therefore, the essence of compulsive drug-seeking behavior and relapse is dysfunctional inhibitory control. Thus, there is a strong association between executive dysfunction and relapse.

2. The age of first MA use is associated with relapse.

Compared to adults diagnosed with MUD whose onset of MA use occurred in adulthood, adolescents (19 years of age or younger) diagnosed with MUD whose onset of MA use occurred in adolescence have displayed less cortical thickness in the prefrontal cortex, which was associated with worse performance on neuropsychological tests assessing executive function (31). This study also showed that the rate of first MA use occurred at 19 years old or younger was positively correlated with the number of relapse. In addition, an earlier onset of adolescent MA use has been related to more metabolic dysfunction in the anterior cingulate cortex and greater deficits in inhibitory control (32). Given that executive dysfunction (including inhibitory control deficits) may be a primary factor influencing drug relapse (12, 17, 33), these previous findings may help explain why age of first MA use and executive dysfunction were associated with MA relapse in the current study.

Furthermore, during adolescence, developmental changes occurring during the maturation of the nervous system lead to increased plasticity in the striatum, resulting in a high density of striatal dopamine receptors, and enhancing susceptibility to MA abuse (34, 35). MA is a drug that mainly acts on the dopamine system, increasing dopamine release to the striatum through mesolimbic pathways (36). Therefore, in adolescents, MA will cause higher levels of excitement and potential damage to the striatum than in adults. The striatum is closely linked to both MA addiction (37) and executive function (38). Therefore, we speculate that this may be one of the reasons why the earlier the age of first MA use, the greater the number of relapses.

However, some researchers suggest that MA has minor damage to cognitive function (39) and some studies even suggest that MA improves cognitive performance in selected domains (40). We suspect that this may be related to the dose and duration of MA use. For example, previous studies have found that short-term administration of MA at low doses can produce neuroprotective effects, but high doses or long-term MA can lead to neurotoxicity (41, 42). In the current study, the executive dysfunction in the MUD patient group that had relapsed once was similar to the executive dysfunction in the MUD patient group without a history of relapse (as shown in Table 4). Still, executive dysfunction in the current study was associated specifically with having relapsed two or more times, which suggests that executive dysfunction may play a role in repeated relapse and thus more chronic use of MA.

This study also found an interesting phenomenon, namely, spearman correlation analyses showed that a significant association between the age of first MA use and the total duration of MA use (*P* < 0.01), in other words, the earlier a person starts using MA, the longer they are likely to use it. In addition, it was also found that both the age of first MA use and the total duration of MA use were associated with the number of relapses (*P* < 0.01 and *P* < 0.05, respectively). However, regression analysis indicated that the total duration of MA use may be less associated with relapse than executive dysfunction and the age of first MA use. Possible reasons are as follows: (1) Relapse after withdrawal from MA use may cause more serious nerve damage than continuous use of the MA. Studies have found that preconditioning with low doses of MA can reduce the neurotoxicity of large doses given later (41, 42). This suggests that relapse after long-term withdrawal may result in the same level of neurotoxicity and cognitive dysfunction as naive drug use, both of which are more serious than long-term continuous drug use. (2) The earlier a person takes drugs, the more likely they are to relapse. Previous studies have found that adolescents are at great risk of starting drug use and subsequent addiction (43). Early drug use, for example, in adolescence, is associated with a greater likelihood of transition from drug use to abuse, leading to dependence, a higher frequency of relapse throughout the life cycle, and a shorter time window from first use to the establishment of dependency (44).

To sum up, the above studies suggest that both the age of first MA use and executive dysfunction are more strongly correlated with the number of relapses than the total duration of MA use. Another reason may be the cross-sectional design which hinders the collection of temporal evidence.

### Limitations

The current study has a number of limitations worth noting. First, this study used a cross-sectional design, which prevents establishing the temporal precedence of executive dysfunction and restricts the ability to make causal inferences. Although executive dysfunction may be secondary to chronic MA use, individuals with lower levels of preexisting executive function may also be more prone to develop and persist in the problematic use of MA. Second, the MUD group consisted of MUD patients in forced isolation as part of their treatment. This forced isolation may exert psychological stress on MUD patients, which might lead to detrimental changes in mental health and executive function. Consequently, this was a potential confounding factor in the present study. Third, because there were only male MUD patients in the Bengbu Compulsory Isolated Drug Rehabilitation Center, we could only recruit male participants for the present study. Therefore, current findings may not generalize to female MUD patients, and additional research including female MUD patients is needed. Fourth, the questionnaire-based (subjective) assessment of executive dysfunction may have been prone to subject and experimenter bias. Future research on MA relapse would benefit from administering more objective neuropsychological assessments, such as the Wisconsin Card Sorting Test (45), event-related potential (46), and eye tracking (47).

Fifth, we did not assess whether MUD was mild, moderate or severe. The severity of MUD was also a potential confounding factor affecting the results of data analysis. Lastly, the present study only included individuals who had been in treatment for < 3 months, and prior research (48) has evidenced that MUD-induced cognitive control deficits may improve with long-term abstinence. Thus, executive dysfunction associated with different stages of MA abstinence remains unknown, justifying further investigation.

## Conclusion

Current results evidenced that patients with MUD have worse executive function and mental health, consistent with prior research. Current findings further suggest that executive dysfunction and the age of first MA use may play important roles in MA relapse: More specifically, lower age of first MA use may influence relapse rate in general, while executive dysfunction may influence repeated relapse in particular. These findings add to the literature concerning factors that may increase the risk of relapse in individuals with MUD.

## Data availability statement

The original contributions presented in the study are included in the article/Supplementary material, further inquiries can be directed to the corresponding authors.

## Ethics statement

The studies involving human participants were reviewed and approved by Institutional Review Board (permission number: 2017-53) of Bengbu Medical University. The patients/participants provided their written informed consent to participate in this study.

## Author contributions

L-LM, L-JW, and D-LJ conceived and designed the experiments. X-CZ, H-JT, W-JW, P-PS, LZhu, YW, H-NL, WZ, Y-JW, and J-DL carried out experiments. LZhu, L-LX, XS, and X-CZ analyzed experimental data. W-YS contributed analysis tools. YW and H-SX wrote the first draft of the manuscript. D-LJ provided critical revision of the manuscript for important intellectual content. All authors have materially participated in the manuscript preparation. All authors contributed to the article and approved the submitted version.

## Funding

This project supported by the provincial Natural Science Foundation of Anhui (1908085MH278), Shanghai Key Laboratory of Psychotic Disorders Open Grant (13dz2260500), Program of Bengbu Medical College Science and Technology Development (2020byzd021 and 2020byzd022), Anhui Provincial Education Department Humanities and Social Science Key Project (SK2021A0430), Bengbu Medical College Innovative Training Program for Postgraduate Students (Byycx21025, Byycx21037, and Byycxz21039), Innovative Training Program for Chinese College Students (S202110367098), and Bengbu Medical College Key Laboratory of Addiction Medicine (29-3). All funders didn't interfere in study design, collection, analysis, interpretation, and writing of manuscript.

## Conflict of interest

The authors declare that the research was conducted in the absence of any commercial or financial relationships that could be construed as a potential conflict of interest.

## Publisher's note

All claims expressed in this article are solely those of the authors and do not necessarily represent those of their affiliated organizations, or those of the publisher, the editors and the reviewers. Any product that may be evaluated in this article, or claim that may be made by its manufacturer, is not guaranteed or endorsed by the publisher.
