# Supplementary material for: Associations of executive function and age of first use of methamphetamine with methamphetamine relapse
Source: Front Psychiatry. 2022 Oct 13;13:971825. doi: 10.3389/fpsyt.2022.971825 (PMC9608758; doi:10.3389/fpsyt.2022.971825)
Supplement: Supplementary file 1 [file Data_Sheet_1.docx]

S Table 2. Comparison of psychological characteristics between the MA group and Control group

| Variable | **MA group**  **(n=168)** | **Control group**  **(n=65)** | **t** | ***P*** |
| --- | --- | --- | --- | --- |
| **BRIEF-A** |  |  |  |  |
| Inhibit | 13.04±3.31 | 10.89±3.05 | 4.524 | 0.000*** |
| Shift | 9.08±2.33 | 8.20±2.36 | 2.565 | 0.011* |
| Emotional Control | 15.40±4.05 | 13.55±4.85 | 2.946 | 0.004** |
| Self-Monitor | 9.46±2.60 | 8.00±2.25 | 3.999 | 0.000*** |
| Initiate | 12.07±3.10 | 11.65±3.05 | 0.943 | 0.347 |
| Working Memory | 12.38±3.19 | 11.14±2.94 | 2.726 | 0.007** |
| Plan/Organize | 14.68±3.82 | 13.91±3.86 | 1.387 | 0.167 |
| Task Monitor | 9.16±2.34 | 9.38±2.52 | -0.641 | 0.522 |
| Organization of Materials | 11.65±2.93 | 11.18±3.72 | 1.004 | 0.317 |
| BRI | 46.98±10.89 | 40.65±11.39 | 3.928 | 0.000*** |
| MI | 59.95±13.57 | 57.26±14.20 | 1.337 | 0.183 |
| GEC（BRIEF-A total score） | 106.92±23.54 | 97.91±25.04 | 2.575 | 0.011* |
| **SCL-90** |  |  |  |  |
| Somatization | 21.79±8.05 | 15.26±5.72 | 6.846 | 0.000*** |
| Obsessive compulsion | 20.75±7.03 | 20.35±5.79 | 0.407 | 0.685 |
| Interpersonal sensitivity | 15.79±5.82 | 15.86±6.34 | -0.079 | 0.937 |
| Depression | 23.66±8.72 | 20.57±8.09 | 2.459 | 0.015* |
| Anxiety | 17.32±6.68 | 15.11±6.11 | 2.305 | 0.022* |
| Hostility | 10.70±4.64 | 8.72±3.26 | 3.127 | 0.002** |
| Phobic anxiety | 9.72±3.51 | 10.12±3.92 | -0.759 | 0.448 |
| Paranoid ideation | 9.76±3.74 | 9.00±3.24 | 1.434 | 0.153 |
| Psychoticism | 16.40±6.08 | 15.23±5.86 | 1.324 | 0.187 |
| Additional items | 12.92±4.35 | 10.06±3.90 | 4.602 | 0.000*** |
| GSI （SCL-90 total score） | 158.66±50.94 | 158.66±46.71 | 2.525 | 0.012* |
| **BIS-11** |  |  |  |  |
| Motor impulsiveness | 40.01±19.21 | 31.96±17.16 | 2.953 | 0.003** |
| Attentional impulsiveness | 44.55±16.61 | 35.15±12.63 | 3.161 | 0.002** |
| Non-planning impulsiveness | 49.14±19.38 | 42.23±15.05 | 2.888 | 0.004** |
| BIS-11 total score | 44.57±15.08 | 37.45±12.27 | 3.395 | 0.001** |
| **AQ-CV** |  |  |  |  |
| Physical Aggression | 46.13±23.09 | 25.66±14.99 | 7.949 | 0.000*** |
| Verbal Aggression | 41.85±20.21 | 25.54±16.73 | 5.783 | 0.000*** |
| Anger | 42.66±23.57 | 30.32±19.34 | 4.099 | 0.000*** |
| Hostility | 26.70±18.14 | 31.76±18.99 | -1.884 | 0.061 |
| Self-Aggression | 30.27±20.98 | 23.38±16.49 | 2.639 | 0.009** |
| AQ-CV total score | 37.52±17.63 | 27.33±14.30 | 4.558 | 0.000*** |

Data accord with normal distribution were given as Mean ±standard deviation (M±SD).

BRIEF-A, Behavior Rating Inventory for Executive Function of adult version; SCL-90, Self-report symptom inventory, Symptom checklist 90; GEC：Global Executive Composite; GSI：Global Severity Index; BIS-11, Barratt Impulsiveness Scale-11; AQ-CV , Chinese version of Buss-Perry aggression questionnaire

**P* < 0.05, ***P* < 0.01, ****P* < 0.001

**S** Table 3. Relationship between MA use history and psychological characteristics of MUD patients

| Variable | Number of relapses（times） | The age of first MA use（years） | Total duration of MA use (months) | MA use before abstinence (g/occasion) |
| --- | --- | --- | --- | --- |
|  | **r** | **r** | **r** | **r** |
| Age（years) | 0.118 | 0.694** | 0.303** | 0.012 |
| Marital status | 0.073 | -0.193* | 0.114 | 0.104 |
| Education year（years） | 0.080 | -0.215** | 0.009 | 0.023 |
| Number of relapses（times） | 1 | -0.274*** | 0.187* | 0.118 |
| The age of first MA use（years） | -0.274*** | 1 | -0.234** | -0.219** |
| Total duration of MA use (months) | 0.187* | -0.234** | 1 | 0.212** |
| MA use before abstinence (g/occasion) | 0.118 | -0.219** | 0.212** | 1 |
| **BRIEF-A** |  |  |  |  |
| Inhibit | 0.139 | -0.152 | 0.097 | 0.145 |
| Shift | 0.096 | -0.013 | 0.075 | -0.023 |
| Emotional Control | 0.146 | -0.078 | 0.129 | 0.056 |
| Self-Monitor | 0.116 | -0.062 | 0.005 | 0.122 |
| Initiate | 0.171 | -0.086 | 0.099 | 0.058 |
| Working Memory | 0.100 | 0.006 | 0.094 | 0.167* |
| Plan/Organize | 0.104 | -0.077 | 0.065 | 0.086 |
| Task Monitor | 0.174 | -0.033 | 0.101 | 0.069 |
| Organization of Materials | 0.149 | -0.043 | 0.128 | 0.045 |
| BRI | 0.145 | -0.093 | 0.095 | 0.089 |
| MI | 0.156 | -0.055 | 0.109 | 0.099 |
| GEC （BRIEF-A total score） | 0.225 | -0.075 | 0.106 | 0.098 |
| **SCL-90** |  |  |  |  |
| Somatization | 0.015 | 0.003 | 0.140 | -0.013 |
| Obsessive compulsion | 0.007 | 0.041 | 0.083 | 0.007 |
| Interpersonal sensitivity | 0.059 | 0.029 | 0.070 | -0.009 |
| Depression | 0.007 | 0.093 | 0.116 | -0.062 |
| Anxiety | 0.004 | 0.127 | 0.038 | -0.026 |
| Hostility | 0.071 | 0.014 | 0.017 | 0.019 |
| Phobic anxiety | 0.059 | -0.016 | 0.020 | 0.104 |
| Paranoid ideation | 0.190* | 0.026 | 0.035 | -0.014 |
| Psychoticism | 0.037 | 0.121 | 0.091 | -0.067 |
| Additional items | 0.075 | 0.039 | 0.047 | -0.051 |
| GSI （SCL-90 total score） | 0.034 | 0.058 | 0.098 | -0.010 |
| **BIS-11** |  |  |  |  |
| Motor impulsiveness | 0.057 | 0.231** | 0.091 | 0.127 |
| Attentional impulsiveness | 0.062 | -0.068 | 0.002 | 0.050 |
| Non-planning impulsiveness | 0.080 | -0.069 | 0.099 | 0.089 |
| BIS-11 total score | 0.155* | -0.153* | 0.083 | 0.111 |
| **AQ-CV** |  |  |  |  |
| Physical Aggression | 0.145 | -0.124 | 0.142 | 0.101 |
| Verbal Aggression | 0.061 | -0.008 | 0.098 | -0.035 |
| Anger | 0.010 | -0.097 | 0.108 | 0.102 |
| Hostility | 0.056 | -0.094 | -0.004 | 0.105 |
| Self-Aggression | 0.163^*^ | 0.014 | 0.129 | 0.043 |
| AQ-CV total score | 0.148 | -0.076 | 0.118 | 0.077 |

MUD，methamphetamine use disorder. BRIEF-A, Behavior Rating Inventory for Executive Function of adult version; BIS-11, Barratt Impulsiveness Scale-11; GEC：Global Executive Composite; GSI：Global Severity Index; SCL-90, Self-report symptom inventory; AQ-CV , Chinese version of Buss-Perry aggression questionnaire. * *P*﹤0.05，** *P*﹤0.01，Bonferroni's corrections（***P<0.05/21=0.0023）

S Table 4. Comparison of demographic information and psychological characteristics in MUD patients with different number of relapses

|  | Number of relapses | | |  | | |
| --- | --- | --- | --- | --- | --- | --- |
|  | Zero^1^（n=46） | Once^2^（n=73） | Twice and more relapse^3^（n=49） | F/ x^2^ | *P* | Post hoc |
| Age（years) | 33.23±7.80 | 34.23±5.74 | 35.12±6.48 | 0.955 | 0.387 |  |
| Education year（years） | 6.89±3.72 | 6.59±2.33 | 7.51±2.63 | 1.392 | 0.251 |  |
| Marital status | 1.52±0.69 | 1.73±0.85 | 1.87±0.94 | 1.569 | 0.199 |  |
| The age of first MA use（years） | 28.5±8.73 | 25.52±5.73 | 23.15±7.07 | 6.570 | 0.002** | 1>2，1>3 |
| First MA use occurred at 19 years old or younger (%) | 6（13.04） | 12（16.43） | 22（44.89） | 17.138 | 0.000** | 3>2，3>1 |
| Total duration of MA use (months) | 102.23±74.68 | 114.48±65.80 | 140.94±75.33 | 3.661 | 0.028* | 1<3，2<3 |
| MA use before abstinence (g/occasion) | 0.48±0.32 | 0.43±0.43 | 0.58±0.32 | 2.199 | 0.114 |  |
| **BRIEF-A** |  |  |  |  |  |  |
| Inhibit | 12.70±2.80 | 12.79±3.42 | 13.94±3.65 | 2.150 | 0.120 |  |
| Shift | 9.13±2.20 | 8.62±2.06 | 9.72±2.76 | 3.288 | 0.040 |  |
| Emotional Control | 15.22±3.58 | 14.70±3.89 | 16.79±4.52 | 3.995 | 0.020 |  |
| Self-Monitor | 9.46±2.58 | 8.94±2.39 | 10.26±2.82 | 3.717 | 0.026 |  |
| Initiate | 11.67±3.16 | 11.93±2.93 | 13.15±3.67 | 2.918 | 0.057 |  |
| Working Memory | 12.10±3.18 | 12.22±3.15 | 12.96±3.31 | 1.018 | 0.364 |  |
| Plan/Organize | 14.46±3.53 | 14.38±3.90 | 15.51±3.97 | 1.398 | 0.250 |  |
| Task Monitor | 8.96±2.37 | 8.93±2.26 | 10.09±2.64 | 3.807 | 0.024 |  |
| Organization of Materials | 11.28±3.00 | 11.41±2.74 | 12.45±3.09 | 2.364 | 0.097 |  |
| BRI | 46.50±9.62 | 45.04±10.30 | 50.70±12.45 | 4.027 | 0.020 |  |
| MI | 58.48±13.15 | 58.88±13.27 | 64.15±14.45 | 2.702 | 0.070 |  |
| GEC （BRIEF-A total score） | 104.9±21.88 | 103.85±22.55 | 114.85±25.65 | 3.857 | 0.023* | 1<3，2<3 |
| **SCL-90** |  |  |  |  |  |  |
| Somatization | 22.16±8.70 | 21.46±7.56 | 21.93±8.26 | 0.116 | 0.891 |  |
| Obsessive compulsion | 21.50±6.30 | 20.25±7.26 | 20.80±7.41 | 0.379 | 0.685 |  |
| Interpersonal sensitivity | 16.16±5.6 | 15.28±5.84 | 16.21±5.10 | 0.548 | 0.579 |  |
| Depression | 24.80±8.93 | 22.94±8.6 | 23.65±8.19 | 0.728 | 0.485 |  |
| Anxiety | 18.68±7.69 | 16.29±6.18 | 17.57±6.25 | 2.054 | 0.132 |  |
| Hostility | 11.00±4.80 | 9.94±4.13 | 11.54±5.11 | 1.697 | 0.187 |  |
| Phobic anxiety | 9.86±3.68 | 9.43±3.03 | 10.00±4.02 | 0.464 | 0.629 |  |
| Paranoid ideation | 10.72±4.43 | 8.99±3.17 | 10.00±3.66 | 3.079 | 0.049* | 2<1 |
| Psychoticism | 17.07±6.01 | 15.68±6.03 | 16.84±6.21 | 0.907 | 0.406 |  |
| Additional items | 13.23±4.79 | 12.42±3.93 | 13.39±4.51 | 1.021 | 0.363 |  |
| GSI （SCL-90 total score） | 162.98±51.59 | 154.18±50.23 | 160.88±42.75 | 0.541 | 0.583 |  |
| **BIS-11** |  |  |  |  |  |  |
| Motor impulsiveness | 41.52±20.27 | 36.40±17.32 | 44.36±20.09 | 2.701 | 0.070 |  |
| Attentional impulsiveness | 45.11±14.76 | 41.81±16.61 | 47.77±17.08 | 1.973 | 0.142 |  |
| Non-planning impulsiveness | 49.67±19.10 | 45.82±19.74 | 53.72±17.63 | 2.502 | 0.085 |  |
| BIS-11 total score | 43.58±13.29 | 41.80±14.79 | 48.62±15.33 | 3.654 | 0.028* | 2<3 |
| **AQ-CV** |  |  |  |  |  |  |
| Physical Aggression | 45.73±22.46 | 41.54±21.84 | 54.56±23.77 | 4.786 | 0.010 |  |
| Verbal Aggression | 43.80±22.07 | 37.47±18.99 | 47.02±19.33 | 3.552 | 0.031 |  |
| Anger | 45.92±21.11 | 38.13±23.18 | 46.45±25.80 | 2.431 | 0.091 |  |
| Hostility | 27.87±18.41 | 23.68±16.57 | 30.55±19.84 | 2.185 | 0.116 |  |
| Self-Aggression | 26.85±18.48 | 28.63±20.59 | 35.96±23.37 | 2.588 | 0.078 |  |
| AQ-CV total score | 38.04±15.86 | 33.96±16.68 | 42.91±19.62 | 3.421 | 0.035* | 2<3 |

One-way ANOVA or Fisher's exact tests with Bonferroni post hoc analysis, test level after correction for multiple comparisons is P=0.05/3=0.017. Only the age of first MA use were significantly different with different number of relapses (P < 0.017). * *P* ﹤0.05; ** *P* ﹤0.017

MUD，methamphetamine use disorder；BRIEF-A, Behavior Rating Inventory for Executive Function of adult version; GEC：Global Executive Composite; SCL-90, Self-report symptom inventory, Symptom checklist 90; GSI：Global Severity Index; BIS-11, Barratt Impulsiveness Scale-11; AQ-CV , Chinese version of Buss-Perry aggression questionnaire
